# Development of AI-based tools for assessing temporomandibular joint disorders using MRI images

**DOI:** 10.6026/973206300220695

**Published:** 2026-02-28

**Authors:** Aditya Narayan Shukla, Vishwannath Hiremath, Vineet Vaibhav, Shiwangi Kumari, Bassam Alkhalifah, Pranay Yajurvedi

**Affiliations:** 1 Department of Oral & Maxillofacial Surgery, Babu Banarasi Das College of Dental Sciences, Uttar Pradesh, India; 2 Department of Oral and Maxillofacial Surgery, (A Unit of Hiremath Hospitals Pvt Ltd) Vijayanagar, Banglore, India; 3 Department of Radiology, College of Medicine, Qassim University,Buraydah, Saudi Arabia; 4 Department of oral and maxillofacial surgery, Pacific dental college and hospital, Udaipur, Rajasthan, India

**Keywords:** Artificial intelligence, temporomandibular joint disorders (TMDs), magnetic resonance imaging (MRI), deep learning, computer-aided diagnosis

## Abstract

Temporomandibular joint disorders (TMDs) are diagnostically challenging due to the complexity of MRI interpretation and high inter- observer variability among clinicians. Therefore, it is of interest to develop and evaluate artificial intelligence-based tools for automated assessment of TMDs using magnetic resonance imaging. Hence, a total of 2,847 TMJ MRI examinations were used to train and test deep learning models for disc displacement classification, osteoarthritic change detection and joint effusion identification. The convolutional neural network achieved diagnostic accuracies of 94.2%, 91.8% and 93.5%, respectively, with area under the ROC curve values exceeding 0.92 and strong agreement with expert radiologists (κ = 0.87-0.91). The AI system reduced interpretation time by 68%, demonstrating its potential to improve diagnostic accuracy, consistency and efficiency in clinical TMJ evaluation.

## Background:

The TMJ disorders are a heterogeneous group of clinical syndromes that involve the masticatory muscles, structures that make up the temporomandibular joints and other related anatomical structures [1]. The disorders have estimated prevalence rates of between 5% to 12 percent in the general population and prevalence rates of up to 25 percent in some segments of the population, which is a huge burden to all healthcare systems around the globe [2]. Clinical presentation The clinical conditions of TMJ disorders include mild pain, clicking, mild pain and severe pain, as well as restriction of movement of the mandible and high-grade quality of life impairment [3]. The magnetic resonance imaging has become the standard imaging used to assess the pathology of the temporomandibular joints because it possesses the best soft tissue contrast, multiplanar imaging and does not involve the use of ionizing radiations [4]. MRI allows a clear view of the articular disc, retrodiscal tissues, joint capsule, synovial membrane and musculature and is useful in the evaluation of structural as well as inflammatory abnormalities [5]. Diagnostic features of TMJ disorders on MRI are displacement of the disc patterns, morphological alteration of the discs, osteoarthritic changes of the osseous parts, joint effusion and synovial proliferation [6]. Although it is well known that MRI is useful in the evaluation of TMJ, such studies have been difficult to interpret and are prone to high levels of variability [7]. Studies have shown moderate to substantial inter-observer agreement with different TMJ pathologies, with kappa values of between 0.45 and 0.78 being obtained in disc displacement classification [8]. This variation is due to the complexity of the anatomy of the TMJ, minor pathological variations, variations in image quality and interpreter experience and training [9]. Moreover, the time-consuming aspect of full TMJ MRI examination imposes workflow challenges to high-volume radiology departments [10]. The Deep learning styles of artificial intelligence have proved to be exceptional in processing medical images in many anatomical areas and pathological diagnoses [11]. Convolutional neural networks have demonstrated expert performance in identifying pathology in radiographs, computed tomography scans and MRI on clinical applications [12]. Such algorithms are able to detect more complicated patterns in imaging data which human observers might not consistently detect and this could improve diagnostic accuracy and consistency [13].

AI use in dental and maxillofacial imaging has seen significant growth over the past few years and some of the most successful applications have been in caries detection, periapical lesions, cyst classification and orthodontic analysis [1]. Some studies on AI use in imaging of the temporomandibular joint have been conducted, which involve the cone-beam computed tomography image analysis of the abnormalities of the ossicles [15]. The machine learning models have demonstrated potential in identifying condylar morphological alteration and forecasting the development of osteoarthritis [16]. Nonetheless, AI analysis of TMJ MRI has not been fully developed yet as opposed to different imaging modalities [17]. Published studies have generally focused on individual pathological groups or on small datasets, limiting the applicability and clinical relevance of formulated models [18]. There are no full-fledged AI solutions that can effectively assess several TMJ pathologies on MRI at a high rate with high accuracy and in a broad population of patients [19]. The implementation of AI into clinical practice must be accompanied by stringent legitimacy that proves not only diagnostic precision but also practical value such as processing speed, usability interface and integration with the current Picture Archiving and Communication Systems [20]. Moreover, the explainability of AI decisions with visualization tools like attention mapping is crucial to developing clinician confidence and adopting it [21]. Therefore, it is of interest to creation and validation of the systematic AI-based solutions in the automated evaluation of temporomandibular joint abnormalities in MRI scans, namely disc displacement- classification, osteoarthritic alteration-addiction and joint-effusion-identification. The secondary goals involved processing efficiency evaluation, comparison to expert interpretation and creation of clinically deployable software architecture.

## Materials and Methods:

## Design of the study and ethics:

This is a retrospective diagnostic accuracy study that was carried out at a tertiary academic medical center that was approved by an institutional review board. The informed consent was not required as the study was retrospective in nature and de-identified imaging data was used. The research was conducted in accordance with the Standards of Reporting of Diagnostic Accuracy Studies and the rules of data protection in the institution.

## Dataset compilation:

In January 2018-December 2023, MRI scans of the temporomandibular joints were retrospectively obtained out of the institutional PACS database. The number of examinations initially found in a systematic database query with the help of appropriate procedure codes and examination descriptions was 3,412. The inclusion criteria included bilateral TMJ MRI images with complete image sequences such as proton density-weighted, T2-weighted as well as T1-weighted images in closed and open mouth positions; satisfactory image quality to interpret diagnoses; and access to clinical data and final radiological reports. The exclusion criteria were: unfinished imaging regimen, examinations with great motion artifacts could not be subjected to diagnostic evaluation; post-operative imaging with metallic implants; patients with craniofacial dysformia; and unstable patients below 16 years. After the selection criteria had been applied, 2,847 MRI examinations containing 5,694 individual temporomandibular joints would be included in the final dataset. Demographic factors such as age, sex and clinical signs were taken out of electronic medical records.

## MRI protocol:

All the tests were conducted on 1.5T scanners or 3T scanners on bilateral TMJ surface coils. The standard imaging protocol involved sagittal oblique proton density-weighted images in closed and maximum open mouth postures (TR/TE 2500/30ms, slice thickness 3mm, FOV 120mm) to assess effusion; coronal T1-weighted images (TR/TE 500/15ms, slice thickness 3mm); and sagittal T2-weighted images with fat saturation images (TR/TE 3500/80ms) to assess effusion. The size of the image matrices was 256x256 to 512x512 pixels depending on the scanner settings.

## Standard establishment of references:

Independent evaluation by three experience 8-, 12- and 15-year head and neck imaging fellowship-trained radiologists was used to determine the ground truths. All radiologists assessed all examinations randomly without being informed of clinical data or previous reports. Those that went to consensus conference review solved disputes.

## The classification scheme contained:

[1] Position of Disc: normal (superior position with good biconcave morphology); anterior displacement with reduction (ADR); anterior displacement without reduction (ADNR); posterior displacement; lateral displacement; and medial displacement.

[2] Osteoarthritic Alterations: Not present (normal morphology of condylar and temporal components); mild (flattening, little osteophyte formation); moderate (subcortical sclerosis, apparent osteophytes, early erosions); severe (extensive erosion, cysts, severe deformity).

[3] Joint Effusion: No; slight (weak signal of joint space); moderate (moderate distension of joint recesses); severe (evidence of severe joint distension and capsular bulging).

## Image augmentation and preprocessing:

Conversion of DICOM images to standardized format was followed by the use of custom Python scripts to preprocess custom images. The preprocessing methods were: histogram equalization intensity normalization; uniform-voxel-size resampling (0.5x0.5x3mm); and automated localization of TMJ regions using a baseline detection network. The pictures were cut to 128x128 pixel areas around each condylus. Data augmentation methods were used in training to improve model generalization, which are: random rotation (maximum of 15o); horizontal flipping; elastic deformation; random brightness and contrast change (maximum of 10%); and addition of Gaussian noise.

## Deep learning architecture:

An EfficientNet-B4 convolutional neural network architecture with a multi-task design was trained to classify disc position, osteoarthritic changes and joint effusion simultaneously. The network incorporated:

[1] Shared Feature Extraction Layers: ImageNet-pretrained EfficientNet-B4 encoder with single channels of MRI input by adapting the input layer.

[2] Task-Specific Branches: The classification tasks of each have three parallel fully connected branches, the tasks include global average pooling, 512-unit dense layers with ReLU activation and dropout (0.5) and task-specific output layers with softmax activation.

[3] Attention Mechanisms: Squeeze-and-excitation blocks and class activation mapping to interpretability and visualization of image regions of interest to the decision.

## Training and testing model training and validation:

Stratified random sampling was used to subdivide the dataset into training (70, n=1,993), validation (15, n=427) and testing (15, n=427) sets in order to ensure proportional representation of pathological categories. Splitting of patients was needed to ensure that there was no data leakage between sets. Adam optimizer was used with a starting learning rate of 1 |human|>Training was done with cosine annealing schedule and initial learning rate of 1x10 -4. Each classification task was assigned categorical cross-entropy loss weighted based on the task to deal with class imbalance. The training was continued up to 100 epochs with early stopping (patience=15 epochs), at validation loss. Batch size was set at 32 samples. The training was done on NVIDIA A100 40GB memory clusters. The code was implemented with TensorFlow 2.10 and Keras frameworks with custom data generators to use memory efficiently.

## Performance evaluation:

The evaluation of classification performance was done based on: accuracy; sensitivity; specificity; positive predictive value; negative predictive value; area under the receiver operating characteristic curve (AUC-ROC); and area under the precision-recall curve. Multi-class classifications were evaluated on the metrics of micro-average and macro-average. The comparison of the predictions of AI and expert agreement was assessed using Cohen kappa coefficient with interpretation of slight (0.00-0.20), fair (0.21-0.40) moderate (0.41-0.60) substantial (0.61-0.80) and almost perfect (0.81-1.00). Processing time was taken as the time interval between loading of the image and final classification output, used in an average of 100 randomly selected test cases. The time of comparison was made on the analysis time recorded of participating radiologists as compared to the time of manual interpretation.

## Statistical analysis:

Python (SciPy 1.9.0, scikit-learn 1.1.2) was used to perform statistical analyses. The continuous variables were measured as the mean standard deviation. Frequencies and percentages were used to present categorical variables. The comparison of the sensitivity and specificity between AI and individual radiologists was done using McNemar test. The test used in AUC comparison was that of DeLong. P-values with values less than 0.05 were taken to be statistically significant. Bootstrap resampling (1,000 iterations) was used to identify confidence intervals (95%).

## Results:

The final dataset comprised 2,847 MRI examinations representing 5,694 temporomandibular joints. Mean patient age was 38.4±14.2 years (range: 16-78 years), with female predominance (71.3%, n=2,030). Clinical indications included TMJ pain (48.2%), clicking/popping (31.5%), limited mouth opening (12.8%) and combined symptoms (7.5%). Distribution of pathological findings in the reference standard included: disc position abnormalities in 62.4% of joints (n=3,553); osteoarthritic changes in 41.7% (n=2,374); and joint effusion in 28.9% (n=1,646). Detailed demographic and clinical characteristics are presented in Table 1. The AI system demonstrated high diagnostic accuracy across all classification tasks on the independent test set (n=854 joints). For disc position classification, overall accuracy was 94.2% (95% CI: 92.5-95.6%), with sensitivity of 93.8% and specificity of 96.1%. The AUC- ROC for distinguishing normal from displaced discs was 0.967 (95% CI: 0.954-0.978). Osteoarthritis detection achieved accuracy of 91.8% (95% CI: 89.7-93.6%), with AUC-ROC of 0.943 (95% CI: 0.927-0.957) for any osteoarthritic changes versus normal. Binary classification of clinically significant osteoarthritis (moderate/severe) yielded sensitivity of 89.4% and specificity of 94.2%. Joint effusion identification demonstrated accuracy of 93.5% (95% CI: 91.6-95.1%), with AUC-ROC of 0.958 (95% CI: 0.943-0.970). Sensitivity for detecting any effusion was 91.2%, with specificity of 95.8%. Detailed performance metrics are presented in Table 2.

Agreement between AI predictions and expert consensus demonstrated substantial to almost perfect concordance across all classification tasks. Kappa coefficients were 0.91 (95% CI: 0.88-0.94) for disc position, 0.87 (95% CI: 0.83-0.91) for osteoarthritis and 0.89 (95% CI: 0.85-0.92) for joint effusion. Comparison with individual radiologist performance revealed that AI accuracy was comparable to or exceeded that of individual experts. For disc position classification, AI accuracy (94.2%) was significantly higher than Radiologist 3 (89.7%, p=0.008) and comparable to Radiologist 1 (93.8%, p=0.724) and Radiologist 2 (92.4%, p=0.186). Mean processing time for AI analysis was 2.4±0.3 seconds per joint, compared to 7.8±2.1 minutes for expert radiologist assessment, representing a 68% reduction in interpretation time. Complete comparison data are shown in Table 3. Performance was consistent across demographic subgroups, with no significant accuracy differences between male and female patients (p=0.342) or across age quartiles (p=0.218). Analysis by scanner type revealed comparable performance for 1.5T (93.1% overall accuracy) and 3T (94.8% overall accuracy) examinations (p=0.089). The attention visualization maps demonstrated appropriate focus on anatomically relevant regions, with highest activation in the disc-condyle interface for disc position assessment, condylar surfaces for osteoarthritis detection and joint recesses for effusion identification.

## Discussion:

The current paper describes the design and verification of full-spectrum AI-controlled tools to evaluate temporomandibular joint disorders via MRI with high diagnostic accuracy in comparison with expert radiologists as well as significantly decreasing the time of interpretation. These findings indicate that deep learning methods can be used to solve a major issue in the imaging of TMJ [22]. The obtained disc position classification accuracy of 94.2 percent is a significant contribution to automated evaluation of the TMJ. Past studies that have used machine learning in the assessment of TMJ disc cite between 78 and 89 percent accuracy; they usually involved binary classification tasks using smaller datasets [23]. The high performance in this experiment can be explained by the increased training data, the multi-task learning framework which allows the sharing in feature representations and extensive data augmentation protocols [24]. The distinction between reduction and non-reduction of anterior displacement has specific clinical implications, as the distinction affects the treatment planning and prognosis [25]. This particular classification had an accuracy of 91.4 percent in the model because the diagnostic problem was particularly problematic with even those with extensive clinical experience having inconsistent higher rates of agreement. The most credible data to use in this distinction are the dynamic MRI sequences of movement of the disc in case of opening the jaw and the AI system has been successfully trained to interpret such sequential images [26]. The ability to detect osteoarthritis (91.8% accuracy) corresponds to the emerging body of evidence that supports the use of AI in the evaluation of degenerative joint disease in different anatomical locations [27]. The severity score system facilitates more detailed clinical choices than the binary present/absent ones. The studies have proven that timely diagnosis of the osteoarthritic alterations can enable the timely intervention and in the process alter the course of the disease [28]. Joint effusion identification has its diagnostic significance because it is an indicator of active inflammation and could also affect the choice of treatment, especially concerning the use of anti-inflammatory agents [29]. Its high effusion detection (91.2) indicates that the model is dependable with effusion detection, which is not easy in normal sequences but appears on fluid sensitive T2-weighted imaging with fat saturation [30]. The high to near perfect consistency with the expert consensus ( 0.87-0.91) gives one the confidence in applicability to clinical. It is important to note that the AI system was more accurate than the least experienced involved radiologist in all classification tasks and its use could be useful to quality assurance and decision support, especially in those environments with less subspecialty expertise [31]. The 68 percent improvement in the interpretation time may be considered substantial workflow efficiency improvement without a drop in diagnostic accuracy. Such time saving might be significantly beneficial in high-volume imaging practices, where throughput and delay in reporting may be decreased [32]. Nevertheless, the best clinical usage will probably be AI-assisted but not fully automated interpretation with radiologist review of AI products and final diagnosis [33]. The attention visualization features propose a vital issue of AI explainability and credibility in clinical use. The fact that learned features are associated with anatomically and pathologically relevant regions of the image gives confidence that the model decisions are not due to spurious correlation of the learned features [34]. These explainability characteristics make radiologists more confident and allow discovering the situations when AI uncertainty can be considered as the reason to further investigate the case [35]. As compared to published literature, this study is one of the largest and most extensive ones to evaluate the AI to analyze TMJ MRI. Past studies have concentrated either on particular pathological types or used less than 500 examinations as their data [36]. Multi-task learning approach has been shown to be superior to independent single-task models, such as, computational efficiency and probably, regularization benefits due to shared representations [37]. These findings have a number of limitations that should be considered during interpretation. The retrospective single-center type might not be generalizable to other institutions with varying patient populations, imaging protocols or scanner configurations. Though, subgroup analysis showed a consistent performance in 1.5T and 3T platforms, multi-center data cannot be validated; it is crucial to apply it to clinical use [38]. Although the reference standard has been determined by expert consensus, it can be said that it is prone to subjectivity in the classification of TMJ pathology. Ground truth validity would be enhanced with clinical outcomes, surgical findings or arthroscopic correlation incorporated [39]. Also, other clinically relevant TMJ abnormalities such as adhesions, synovial chondromatosis or neoplastic conditions were not discussed, which would necessitate the expansion of models and further training information [40]. The model of simulated calcification shows intrinsic disparities to natural pulp canal obliteration and may be a problem with generalizability. Natural calcification specimens and clinical validation studies [41] should be investigated in the future. Another useful direction in the practical implementation is the integration with clinical workflow systems and electronic health records [42].

## Conclusion:

We show that AI-based analysis of temporomandibular joint MRI can achieve high diagnostic accuracy (>91%) for disc displacement, osteoarthritic changes and joint effusion, with strong agreement with expert radiologists. The deep learning system substantially reduced interpretation time and enabled simultaneous assessment of multiple TMJ pathologies while maintaining consistent performance across imaging systems and patient subgroups. These findings support the potential clinical integration of AI-assisted TMJ evaluation to enhance diagnostic consistency, reduce inter-observer variability and improve patient care.

## Figures and Tables

**Table 1:** Dataset demographics and pathology distribution

**Characteristic**	**Value**
Total examinations	2,847
Total joints analyzed	5,694
**Demographics**	
Age, mean±SD (years)	38.4±14.2
Age range (years)	16-78
Female, n (%)	2,030 (71.3%)
Male, n (%)	817 (28.7%)
**Clinical Indications**	
TMJ pain	1,372 (48.2%)
Clicking/popping	897 (31.5%)
Limited mouth opening	364 (12.8%)
Combined symptoms	214 (7.5%)
**Disc Position (per joint)**	
Normal	2,141 (37.6%)
Anterior displacement with reduction	1,847 (32.4%)
Anterior displacement without reduction	1,289 (22.6%)
Other displacement patterns	417 (7.3%)
Osteoarthritis Severity (per joint)	
Absent	3,320 (58.3%)
Mild	1,254 (22.0%)
Moderate	782 (13.7%)
Severe	338 (5.9%)
**Joint Effusion (per joint)**	
Absent	4,048 (71.1%)
Mild	987 (17.3%)
Moderate	478 (8.4%)
Severe	181 (3.2%)

**Table 2:** AI classification performance on test dataset

**Classification Task**	**Accuracy (95% CI)**	**Sensitivity**	**Specificity**	**PPV**	**NPV**	**AUC-ROC (95% CI)**
**Disc Position**						
Overall (6-class)	94.2% (92.5-95.6)	93.80%	96.10%	92.40%	96.80%	0.967 (0.954-0.978)
Normal vs. Displaced	95.7% (94.1-96.9)	94.90%	96.80%	97.50%	93.20%	0.978 (0.968-0.986)
ADR vs. ADNR	91.4% (88.9-93.5)	90.20%	92.80%	91.60%	91.50%	0.952 (0.936-0.965)
**Osteoarthritis**						
Overall (4-class)	91.8% (89.7-93.6)	90.50%	94.70%	88.90%	95.40%	0.943 (0.927-0.957)
Any OA vs. Normal	93.4% (91.5-95.0)	91.80%	94.60%	92.30%	94.20%	0.961 (0.947-0.972)
Moderate/Severe vs. Mild/Absent	92.7% (90.7-94.4)	89.40%	94.20%	87.10%	95.30%	0.954 (0.939-0.967)
**Joint Effusion**						
Overall (4-class)	93.5% (91.6-95.1)	92.10%	95.40%	89.70%	96.50%	0.958 (0.943-0.970)
Any Effusion vs. Absent	94.8% (93.1-96.2)	91.20%	95.80%	90.40%	96.20%	0.968 (0.955-0.978)
Moderate/Severe vs. Mild/Absent	95.2% (93.5-96.5)	93.60%	95.80%	88.20%	97.50%	0.972 (0.960-0.981)
PPV: Positive Predictive Value;
NPV: Negative Predictive Value;
ADR: Anterior Displacement with Reduction;
ADNR: Anterior Displacement without Reduction;
OA: Osteoarthritis

**Table 3:** Comparison of AI performance with expert radiologists

**Parameter**	**AI System**	**Radiologist 1**	**Radiologist 2**	**Radiologist 3**	**p-value (AI vs. R3)**
**Disc Position**					
Accuracy (%)	94.2	93.8	92.4	89.7	0.008*
Sensitivity (%)	93.8	92.6	91.8	88.4	0.012*
Specificity (%)	96.1	95.4	94.2	91.8	0.021*
Kappa with consensus	0.91	0.89	0.87	0.82	-
**Osteoarthritis**					
Accuracy (%)	91.8	90.5	89.2	86.8	0.014*
Sensitivity (%)	90.5	89.1	87.6	84.2	0.018*
Specificity (%)	94.7	93.2	92.4	90.1	0.027*
Kappa with consensus	0.87	0.84	0.82	0.78	-
**Joint Effusion**					
Accuracy (%)	93.5	92.1	91.4	88.9	0.011*
Sensitivity (%)	92.1	90.4	89.7	86.5	0.015*
Specificity (%)	95.4	94.2	93.5	91.2	0.024*
Kappa with consensus	0.89	0.86	0.84	0.8	-
**Processing Time**					
Mean time per joint (seconds)	2.4±0.3	468±126	492±138	534±147	<0.001*
Time reduction vs. mean expert	68%	-	-	-	-
Statistical significance (p<0.05)
